# A Case of Dedifferentiated Liposarcoma That Contributes to Accompanying Vessels of Various Size

**DOI:** 10.3390/diagnostics14151679

**Published:** 2024-08-02

**Authors:** Yosuke Yamada, Kai Mizoguchi, Eisuke Shiba, Honami Mishima, Shinya Otsuki, Masahito Hoki, Masahiro Hirata, Akio Sakamoto, Shuichi Matsuda, Alexander Marx, Masanori Hisaoka, Hironori Haga

**Affiliations:** 1 Department of Diagnostic Pathology, Kyoto University Hospital, 54 Shogoin Kawahara-Cho, Sakyo-Ku, Kyoto 606-8507, Japan; 2 Department of Pathology and Oncology, School of Medicine, University of Occupational and Environmental Health, Kitakyushu 807-8555, Japan; 3 Graduate School of Medicine and Faculty of Medicine, Kyoto University, Kyoto 606-8501, Japan; 4 Department of Orthopaedic Surgery, Kyoto University Hospital, Kyoto 606-8507, Japan; 5 Institute of Pathology, University Medical Center Göttingen, University of Göttingen, 37075 Göttingen, Germany

**Keywords:** soft tissue neoplasms, dedifferentiated liposarcoma, cell trans-differentiation, blood vessels, MDM2, aSMA

## Abstract

Dedifferentiated liposarcoma (DDLPS) is a non-lipogenic sarcoma, generally arising from well-differentiated liposarcoma (WDLPS), although it can develop de novo. DDLPS tumors rarely trans-differentiate into non-adipose mesenchymal tissues; however, the latter lack notable variety and mostly show striated muscle or osteogenic/chondrogenic differentiation. Here, we report a case of DDLPS that contained numerous atypical vessels. A man in his sixties presented with a large tumor in his right thigh, and the tumor was surgically resected. Microscopically, most of the tumor was WDLPS, but a minor portion showed DDLPS, consisting of high-grade spindle cells. Remarkably, the DDLPS contained vessels of various sizes with atypical cytoarchitecture, including vessels with seemingly muscular layers. Immunohistochemically, the atypical cells within the vascular wall expressed aSMA, consistent with smooth muscle cells or pericytes, whereas surrounding high-grade spindle cells only focally expressed it, and these aSMA-positive cells within the vessels exhibited *MDM2* amplification by immuno-fluorescence in situ hybridization. Our results demonstrate that DDLPS can trans-differentiate into smooth muscle cells of various-sized accompanying vessels, which may support their survival and proliferation.

Dedifferentiated liposarcoma (DDLPS) is relatively common in adults, especially in the peritoneum. It typically occurs as a non-lipogenic sarcoma resulting from the progression of a well-differentiated liposarcoma (WDLPS), although a well-differentiated component may not be identifiable [1]. *MDM2* amplification and the resultant MDM2 overexpression are hallmark features of both WDLPS and DDLPS [1], and cells within the tumors with these features may be interpreted as tumor-derived cells. Approximately 10% of DDLPS are reported to have heterologous components that almost always exhibit rhabdomyomatous, osteogenic, or chondrogenic differentiation [1]. A male in his sixties visited our hospital because of a tumor in his right thigh. He had noticed the tumor approximately 1 year previously, but it had recently begun to grow rapidly. Magnetic resonance imaging revealed a large (24.0 × 19.0 × 13.0 cm) multi-lobulated tumor with both T1- and T2-hyperintensities, suggesting WDLPS (Figure 1a). Histology (from needle biopsies) confirmed the clinical diagnosis, and the tumor was surgically resected. Macroscopically, the tumor was multi-nodular and yellowish in most parts, consistent with WDLPS. However, it consisted, in part, of tan-colored, non-lipomatous areas containing small black “dots,” suggesting blood vessels (Figure 1b). Microscopically, the yellowish areas exhibited WDLPS, as expected (Figure 1c [upper],d), whereas the tan-colored areas comprised non-lipogenic, high-grade spindle cells; DDLPS was thus diagnosed (Figure 1c [lower]).

The DDLPS contained numerous vessels, which were often congested and thrombotic (Figure 2a). These vessels varied in size, shape, and structure (Figure 2a–e). Larger vessels consisted of relatively flattened innermost cells and thick vascular walls. The vascular wall comprised irregularly distributed spindle or pleomorphic cells in a myxoid or fibrous background, with a lower cell density than the surrounding tissue consisting of morphologically undifferentiated DDLPS tumor cells, and was thus easily discernible (Figure 2b,c). These cells within the vascular wall often had elongated cytoplasms (Figure 2c) and rarely showed mitoses (Figure 2c [inset]). Smaller vessels generally consisted of innermost cells and outer cells with enlarged nuclei and eosinophilic cytoplasms, which were discernible from surrounding tumor cells (Figure 2e).

Considering the peculiar distribution of the vessels and their cytological atypia, we speculated that these atypical vessels might be, at least in part, derivatives of the DDLPS. However, another possibility was that they were entirely non-neoplastic vessels infiltrated by DDLPS tumor cells. To address our hypothesis, we first performed immunohistochemistry (IHC) for ERG (a marker of vascular endothelial cells) and alpha SMA (a marker of vascular smooth muscle cells or pericytes) [1], to evaluate the immunophenotypes of the atypical cells located within the vessels. IHC revealed that the innermost and outer cells in the vessels, irrespective of size (Figure 3a,b: larger vessels, Figure 3c,d: smaller vessels), were positive for ERG (Figure 3a,c) and alpha SMA (aSMA) (Figure 3b,d), respectively, consistent with the immunophenotypes of endothelial and smooth muscle cells (or pericytes), whereas DDLPS tumor cells surrounding the atypical vessels were only focally positive for these markers (Figure 3a–d). If the atypical cells within the vessels were DDLPS tumor cells simply infiltrating the vessels, their phenotypes would be the same as those of the surrounding tumor cells. Thus, these results suggested that the atypical cells within the vessels contributed to (or formed a part of) the vessels. Furthermore, the larger vessels were sometimes positive for desmin in the outermost parts, similar to physiologically large vessels (not shown). Comparing the two cell types, the number of aSMA-positive cells was much greater than the number of ERG-positive cells, and some of the aSMA-positive cells appeared to reach the lumen (Figure 3b,d).

Next, we performed immunohistochemistry (IHC) for CDK4 and MDM2, both of which are expressed in most cases of DDLPS [1]. IHC revealed that the atypical cells, both seemingly endothelial and smooth muscle cells, expressed CDK4 (Figure 4a,c) and MDM2 (Figure 4b,d). These results suggested that the atypical cells within the atypical vessels are DDLPS tumor cells. 

Our immunohistochemistry (IHC) results revealed that (1) the atypical cells within the atypical vessels exhibited reasonable immunophenotypes as vessel-forming cells and that (2) these atypical cells exhibited immunophenotypes of DDLPS tumor cells. Accordingly, we performed chromogenic in situ hybridization (CISH) for *MDM2* and immuno-fluorescence in situ hybridization (immuno-FISH) for (1) CD31 (a marker of vascular endothelial cells) and *MDM2*, and (2) aSMA and *MDM2*, to address whether immunohistochemically confirmed endothelial (CD31-positive) cells and smooth muscle (aSMA-positive) cells exhibit *MDM2* amplification, the gold standard to demonstrate the presence of DDLPS tumor cells [1]; CD31, instead of ERG, was used for these analyses because ERG is a nuclear protein and masks the signals of ISH. CISH demonstrated that both cell types seemed to harbor *MDM2* amplification (Figure 5a–d). Furthermore, immuno-FISH demonstrated that aSMA-positive cells, throughout the vessel walls and irrespective of vessel size, did show *MDM2* amplification (Figure 5e,f [arrow]), demonstrating that these cells are derived from DDLPS tumor cells as a result of trans-differentiation. In contrast, CD31-positive cells with *MDM2* amplification were not evident (not shown). Dedifferentiation and trans-differentiation of dedifferentiated cells are well-known phenomena in liposarcoma. However, the resultant cell types after trans-differentiation are generally limited; they are myogenic, chondrosarcomatous, or osteosarcomatous cells in most cases of DDLPS, although angiosarcomatous trans-differentiation was suggested in one case report [1,2]. Thus, our DDLPS case, in which cells trans-differentiated into and contributed to smooth muscle cells of various-sized vessels within the tumor, is noteworthy because vessels within tumors are generally regarded as non-neoplastic structures [3,4,5]. The contribution of liposarcoma tumor cells to the vascular wall has been proposed by two groups [6,7]. Folpe et al. described WDLPS with an intrinsic smooth muscle component as lipo-leiomyosarcoma and speculated that the smooth muscle component originated from atypical changes in the muscular vessel wall [6]. However, this speculation was based solely on the morphological evaluation of hematoxylin-eosin staining. Shen et al. reported pericyte-like differentiation of small vessels in WDLPS by immunohistochemical and FISH analyses [7]. Our case supports and connects these previous studies and expands the concept of “blood vessel contribution” of liposarcoma to DDLPS. We recognize that *MDM2* amplification was not evident in some cells comprising the atypical vessels; specifically the CD31-positive endothelial cells and the outermost desmin-positive cells of the vessels. Thus, we hypothesize that the atypical vessels were chimeric; that is, they were formed by a combination of tumor cells and non-neoplastic mesenchymal cells. Open questions are (1) whether trans-differentiation into smooth muscle cells consisting of vessels in DDLPS is rare or whether it can be observed in other cases; and (2) whether this type of trans-differentiation has clinical significance. Regarding the first question, in our case, the vessels that the DDLPS cells contributed to were unusual for completely non-neoplastic vessels because, in addition to the atypical cytoarchitecture, they were densely aggregated only within DDLPS areas, and large vessels with thick walls are unlikely to be formed by tumor angiogenesis [3,4,5]. Thus, these macroscopic and microscopic features may lead us to suspect that the vessels consist of tumor cells. Related to the second question, we think that the tumor-derived vessels could connect to the circulatory system because the vessels we observed were often congested by red blood cells. If that is the case, it is not surprising that tumor-derived vessels confer growth and metastatic advantages on the tumor. Larger retrospective studies are warranted to address the above questions. We believe this case may help expand the concept of trans-differentiation and open new avenues for studies on the “potentially self-supporting” cellular plasticity of human neoplasms.

## Figures and Tables

**Figure 1:**
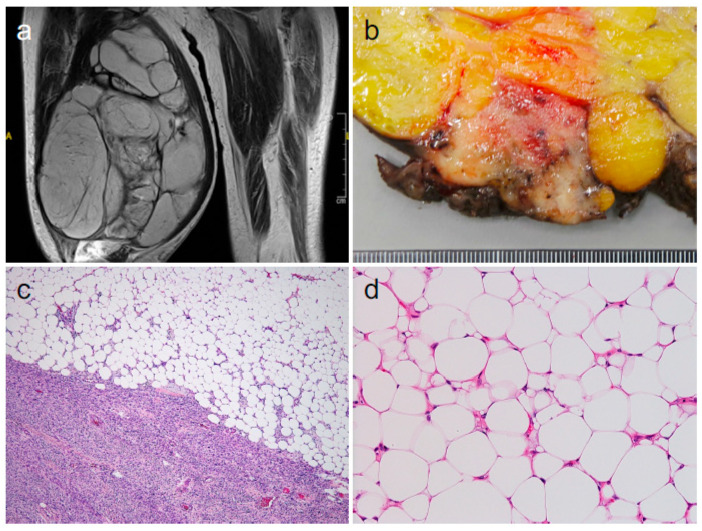
Imaging, macroscopic, and microscopic findings of the tumor. (**a**); T2-weighted imaging, (**b**); gross appearance, ((**c**,**d**); hematoxylin and eosin staining [original magnifications: (**c**), ×20; (**d**), ×200]).

**Figure 2:**
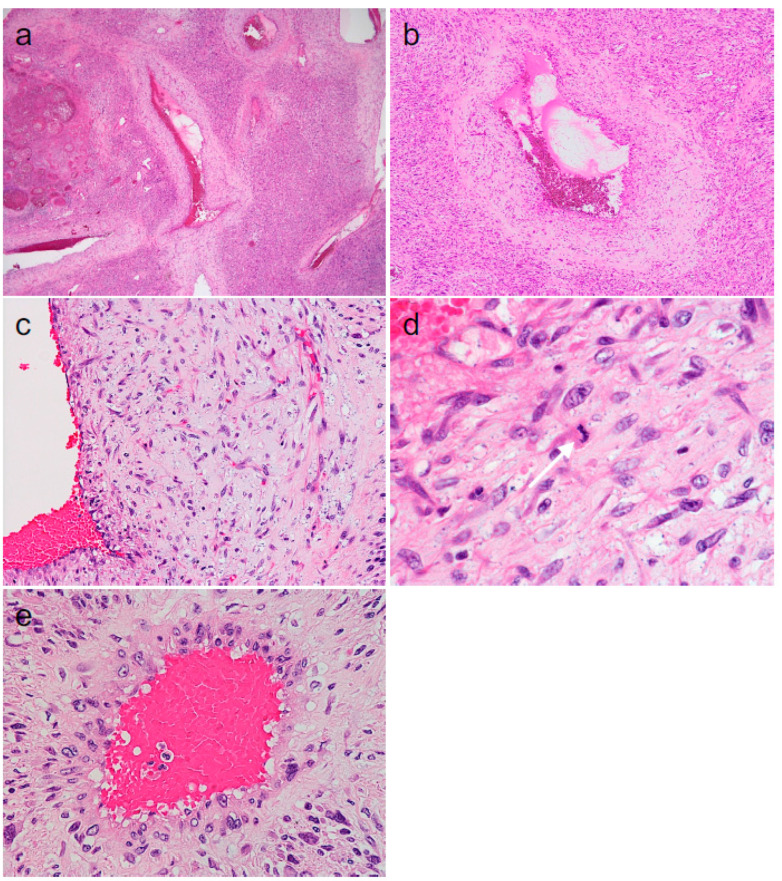
Microscopic findings of dedifferentiated liposarcoma components of the tumor. ((**a**–**e**): hematoxylin and eosin staining [original magnifications: (**a**), ×20; (**b**), ×40; (**c**), ×200; (**d**), ×400]).

**Figure 3:**
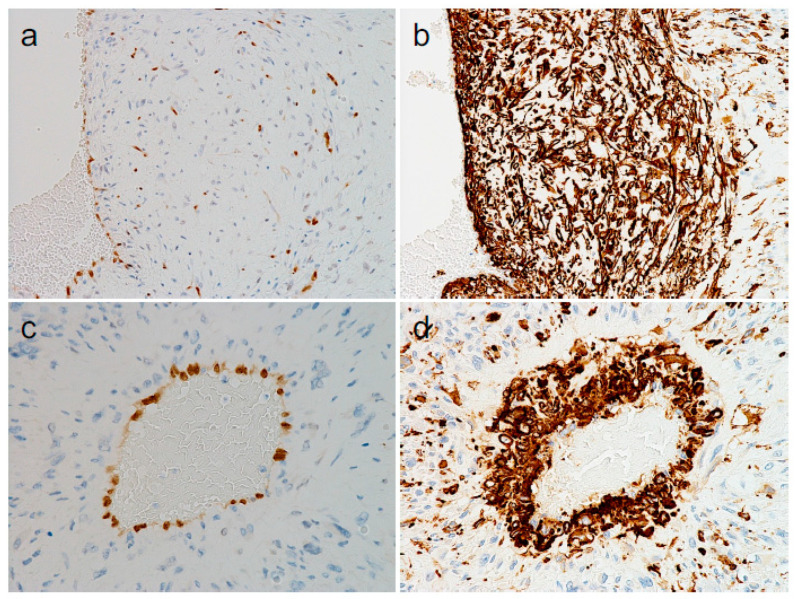
Immunohistochemical findings of atypical vessels within dedifferentiated liposarcoma. ((**a**–**d**): immunohistochemistry for ERG and aSMA [original magnifications: (**a**,**b**), ×200; (**c**,**d**), ×400]).

**Figure 4:**
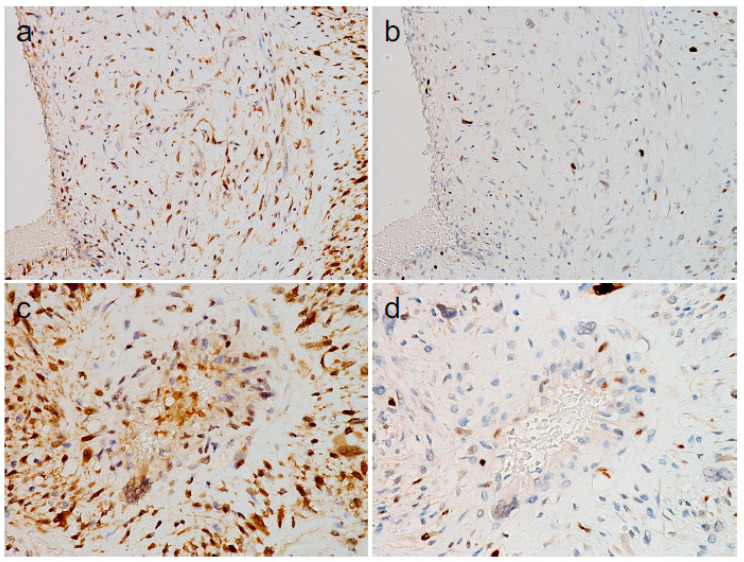
Immunohistochemical findings of atypical vessels within dedifferentiated liposarcoma (continued). ((**a**–**d**): immunohistochemistry for CDK4 and MDM2 [original magnifications: (**a**,**b**), ×200; (**c**,**d**), ×400]).

**Figure 5:**
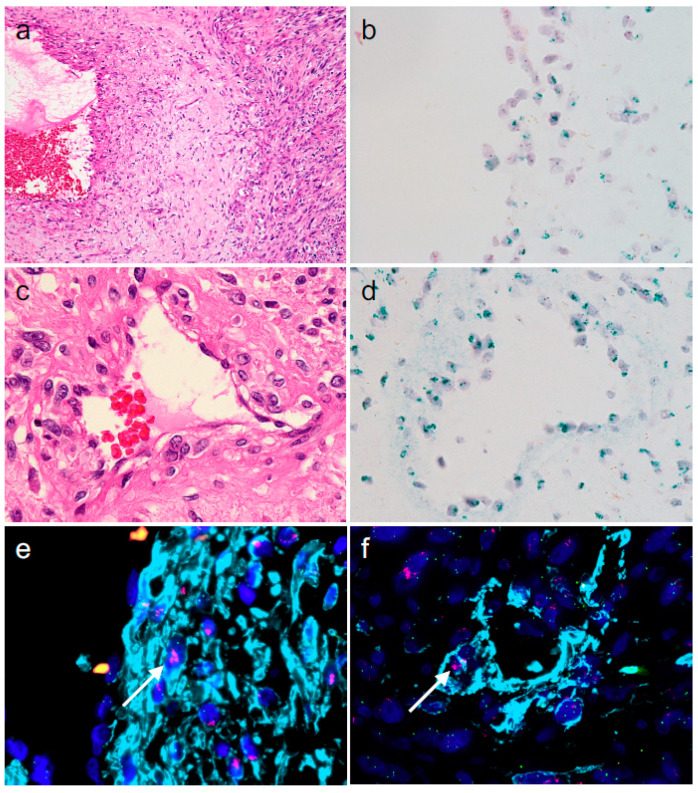
Findings of chromogenic in situ hybridization for MDM2 and immuno-FISH for aSMA and MDM2 in atypical vessels. ((**a**,**c**): hematoxylin and eosin staining; (**b**,**d**): chromogenic in situ hybridization for *MDM2*; green dots = *MDM2*, red dots = *CEN12*; (**e**,**f**): immuno-FISH for *MDM2* and aSMA; red dots = *MDM2*, light blue = aSMA, dark blue = DAPI [original magnifications: (**a**), ×100; (**b**–**f**): ×400]).
